# Supplementary material for: Polyketide synthase gene clusters in the ericoid mycorrhizal fungus Oidiodendron maius: genome-wide prediction, expression profiling, and functional characterization of knockout mutants
Source: Front Plant Sci. 2026 Sep 16;17:1924430. doi: 10.3389/fpls.2026.1924430 (PMC13623903; doi:10.3389/fpls.2026.1924430)
Supplement: Supplementary file 1 [file SupplementaryFile1.zip › Supplementary Materials/Supplementary Folder 1.zip!/Oidma1_AssemblyScaffolds_Repeatmasked/knownclusterblast/cluster55/jgi.p_Oidma1_21636_mibig_hits.html]

| MIBiG Protein | Description | MIBiG Cluster | MiBiG Product | % ID | % Coverage | BLAST Score | E-value |
| --- | --- | --- | --- | --- | --- | --- | --- |
| EAL89348.2 | conserved\_hypothetical\_protein | BGC0001403 | Trypacidin\_biosynthetic\_gene\_cluster | 52 | 93.9759036145 | 152 | 5e-37 |
| CBF90095.1 | conserved\_hypothetical\_protein | BGC0001271 | Emericellin\_biosynthetic\_gene\_cluster | 40 | 110.240963855 | 126 | 2.2e-29 |
| AGO59043.1 | PtaC | BGC0000121 | Pestheic\_acid\_biosynthetic\_gene\_cluster | 43 | 81.3253012048 | 113 | 2e-25 |
